# Top Management Team Intrapersonal Functional Diversity and Adaptive Firm Performance: The Moderating Roles of the CEO–TMT Power Gap and Severity of Threat

**DOI:** 10.3389/fpsyg.2021.772739

**Published:** 2021-12-23

**Authors:** Changlong Ma, Yuhui Ge, Jingwei Wang

**Affiliations:** Business School, University of Shanghai for Science and Technology, Shanghai, China

**Keywords:** top management team, team adaptation, intrapersonal functional diversity, CEO-TMT power gap, severity of threat, adaptive firm performance

## Abstract

While usually argued to be improving firm performance, the effect of top management team (TMT) functional diversity on firm performance is mixed. Bridging the TMT diversity, team adaptation, and threat-rigidity literature, we present a contingency model in which the relationships between intrapersonal functional diversity (at both CEO and TMT levels) and adaptive firm performance depend on the CEO–TMT power gap and severity of threat. To test our hypotheses, 270 firms, which have been severely affected due to the COVID-19 pandemic, were selected from China's A-share listed companies. Multiple regression analyses have shown that a moderation of CEO intrapersonal functional diversity's effect on adaptive firm performance by the CEO–TMT power gap is moderated by the severity of threat. However, no significant main or interaction effect of TMT intrapersonal functional diversity was found. The findings of this study have implications for the recovery or improvement of firm performance in threat situations.

## Introduction

Various crisis episodes like the Chernobyl accident, Wenchuan earthquake, global financial crisis (2008–2009), and COVID-19 epidemic have occurred frequently in recent years, and new crisis episodes may break out at any moment. The old Chinese proverb “In nature, there are unexpected storms and in life, unpredictable vicissitudes” indicates that disasters may occur stochastically and unsteadily, especially in the modern world of Volatility, Uncertainty, Complexity, and Ambiguity (VUCA). Note that the change has become an eternal reality of modern organizations that need to be addressed squarely, adaption has drawn considerable attention in the research field of team effectiveness, and organizations increasingly rely on the capacity of teams to survive (Burke et al., 2006; Rosen et al., 2011; Gevers et al., 2015; Georganta et al., 2021).

Top management team (TMT) is the focus entity of an organizational strategic decision, which determines the survival and development of enterprises to a large extent. According to the Upper Echelons Theory (Hambrick and Mason, 1984), TMT characteristics are associated with managerial knowledge, values, and perceptions and can thus be used to predict organizational outcomes. Therefore, “optimal TMT characteristic composition” has become an important concern to strategic management researchers. Theoretically, heterogeneous TMT has advantages in information processing and can better adapt to internal or external changes. The last few decades have witnessed a rapid growth of the body of studies examining the relationship between TMT diversity and organizational outcomes, and the findings are full of confusion (Hambrick et al., 2015). Although a consensus is emerged to consider internal or external situational factors in reconciling the mixed results (Cannella et al., 2008). Unfortunately, very few studies have been conducted on how teams adapt to challenging or threat situations (Marks et al., 2000; Gevers et al., 2015; Klein and Kozlowski, 2016), especially the moderating role of both internal and external environments. Overall, the demographic characteristics of executives are valid, albeit incomplete and imprecise, proxies of executives' cognitive frames (Hambrick, 2007).

The Team Adaptation Theory could complement the Upper Echelons Theory by focusing on one indicator of the external environment: the nature of the trigger or disruption. For example, a growing body of literature suggests that the effectiveness of the team adaptation process depends on the nature of the adaptation trigger faced by the team (Baard et al., 2013; Maynard et al., 2015; Christian et al., 2017). This constructive recommendation is consistent with the contingency perspective that a team whose internal structure matches its corresponding external demands is more likely to encounter a better performance (Ellis, 2006; Cooper et al., 2014). Therefore, to understand the dynamic mechanism of the confusing impact of TMT composition on various organizational outcomes extracted from the past studies, researchers need to investigate not only the existence of the triggers but also the nature of the triggers such as type (e.g., teamwork based or taskwork based), origin (e.g., internal-resource or external-resource), or duration (e.g., temporary or sustained) of the triggers.

On the other hand, the Team Adaptation Theory also complements the Upper Echelon Theory by emphasizing the role of certain key members of the team in the face of triggers or disruptions (Maynard et al., 2015), such as team member turnover, acute stress, and overseas listing. This is in contrast to the views of the Upper Echelons perspective that pay more attention to the entire TMT (Hambrick and Mason, 1984; Hambrick, 2007). Specifically, considering the uneven distribution of organizational power (Finkelstein, 1992), the powerful actors normally play a more important role in the process of decision-making and decision execution (Hambrick and Mason, 1984). Meanwhile, an adaptation trigger is an event that usually poses a significant threat to the focus entity and with little time to respond. Therefore, it is necessary to distinguish the different roles of these characteristics at the individual level and the team level in theoretical and empirical research.

Based on the above analysis, this article focuses on the question of “optimal TMT characteristic composition” under different combinations of internal and external environments, which is the effect and mechanisms of TMT characteristic diversity on the adaptive firm performance (i.e., the recovery or improvement of the firm performance). From the literature, it is known that functional background is the most common but also the most important form of characteristics studied by TMT researchers. TMT intrapersonal functional diversity, an important but a less studied type of functional diversity developed by Bunderson and Sutcliffe (2002) is considered to be related to the firm performance (Cannella et al., 2008). According to the recommendations of Maynard et al. (2015) mentioned above, this paper focuses on the intrapersonal functional diversity at both CEO and TMT levels under the different situations of severity of threat, and three sub-questions are then proposed: First, CEO with narrower or broader functional experiences, what kind of CEO is more adaptive? Second, what kind of TMT is more adaptive, TMT composed of executives with narrower or broader functional experiences? Third, with different combinations of the internal (i.e., CEO–TMT power gap) and external environment (i.e., severity of threat), what is the relationship between intrapersonal functional diversity and adaptive firm performance at both CEO and TMT levels.

On the research basis of Cannella et al. (2008) and Maynard et al. (2015), the contributions of the study are three-fold: (1) we expand TMT diversity research by confirming the differentiated impact of intrapersonal functional diversity on adaptive firm performance at CEO and TMT levels. (2) Both internal and external environments are considered simultaneously during examining the moderating role of the contextual factors. A negative three-way moderating effect among CEO intrapersonal functional diversity, the CEO–TMT power gap, and the severity of threat provides a more comprehensive lens on exploring the impact of TMT functional diversity on organizational outcomes. (3) The results provide preliminary empirical evidence for the viewpoint of the team adaptation theory that the characteristic of the team leader is more valuable than the average characteristic of the team members in the recovery and improvement of firm performance.

## Literature Review and Hypotheses

### Intrapersonal Functional Diversity and Adaptive Firm Performance

Intrapersonal functional diversity was defined as the breadth of one's functional experience (Bunderson and Sutcliffe, 2002; Cannella et al., 2008). Individuals with great intrapersonal functional diversity mean that the member has worked in more functional areas with a shorter average tenure in each function area. Consequently, the knowledge structure characterized by larger breadth and smaller depth is formulated. According to the Upper Echelons Theory, individuals tend to gather and interpret information based on their prior experiences and expectations to minimize the depletion of the limited cognitive resources (Hambrick and Mason, 1984). This filtering process is similar to the mechanism of Mind-sponge (Vuong and Napier, 2015), which argues that every person has a mindset, value, or belief, which are used as benchmarks to judge the usefulness of information or make decisions and responses. Therefore, CEOs with great intrapersonal functional diversity are usually more open to new experiences and can thus reduce the parochialism that often characterizes the executives of narrow functional experience (Cannella et al., 2008). In other words, an important part of the explanation rests with the phenomenon of “cultural additivity,” which is defined as the arbitrary tolerance of and willingness to add new beliefs, values, or norms even when there was a contradiction, to the existing belief systems, and it appears to be an important antecedent of quick and flexible adoption of, and adaptation to, new ideas (Vuong et al., 2018).

Moreover, individuals have a tendency to classify similar members as in-group members and dissimilar members as out-group members and show a favor for in-group over out-group in evaluations and behaviors (Tajfel and Turner, 1986). Considering that CEOs with high intrapersonal functional diversity usually have a common functional experience with other teammates, they are more likely to show more inclusiveness, and can thus create equal access for TMT strategic decision-making, resources, and upward mobility opportunities for other executives (Martins, 2020). In turn, other executives are more likely to engage in constructive debates and voice behaviors. As a result, better and thorough evaluations of the alternatives can be obtained, and better adaptive firm performance may follow.

However, scholars also suggest that a more thorough exploration of the role of knowledge depth and knowledge width should be conducted. For example, a field study of exploring the knowledge–creativity relationship has found that the effect of knowledge depth and knowledge breadth depends on the career age (Mannucci and Yong, 2018). Specifically, knowledge depth has a positive effect in the early years of the career and negative in later stages. In contrast, knowledge breadth does not have a significant effect in the early years but it has a positive effect in later stages. This means that the knowledge structure with complex and homogeneous characteristics is a double-edged sword. Not only can it provide more chances for recombining the existing knowledge (Gino et al., 2010) but also may lead to cognitive rigidity with a strong linkage between and within knowledge domains, which is likely to impede a new recombination of existing knowledge (Dane, 2010). Therefore, CEOs with high intrapersonal functional diversity may have the advantage of flexibility but also the disadvantage of the reduction of knowledge recombination.

At the team level, shared functional experiences among executives would improve the similarity of TMT task mental models and enable executives to predict each other's behavior more accurately under the conditions that the communication for necessary strategy formulation is difficult. Numerous studies have shown that task mental models are positively related to team performance (Mathieu et al., 2000). Therefore, we argue that TMT intrapersonal functional diversity has a positive effect on adaptive firm performance. In addition, shared functional experiences make it easier for team members to connect with each other. As a result, executives with high intrapersonal functional diversity are more likely to occupy the central position of the functional network, which enables them to control the flow of information across the network. This is also known as the brokerage (Li et al., 2018). In other words, executives with broader functional experiences can translate one individual's perceptions into a form that others can understand and not be redundant (Hambrick and Mason, 1984). Thus, executives with high intrapersonal functional diversity can improve the efficiency and quality of information sharing process between the teammates who are not alike, and a substantial number of studies indicate that knowledge sharing is one of the most important antecedents of team effectiveness (Collins and Smith, 2006; Carmeli and Paulus, 2015).

Of course, TMT intrapersonal functional diversity may also have some drawbacks. As described previously, TMT with high intrapersonal functional diversity is actually a homogeneous team, where each of its members has a broad functional experience (Cannella et al., 2008). This means that even though common experiences may contribute to dysfunctional conflict management, the lessening of the team's knowledge base is inevitable. Along this line, the common experiences may block access to the new information, new explanations, and the new plans in coping with the triggers, and thus impairing decision effectiveness.

In summary, an intrapersonal functional diversity is a double-edged sword characterized by efficiency (dis)advantage and quality (dis)advantage of decision-making. It is improper to predict any main effect of intrapersonal functional diversity on adaptive firm performance at both CEO or the TMT levels.

### Internal Context: The Moderating Role of CEO–TMT Power Gap

A valuable explanation of the mixed TMT diversity-organizational outcome relationship is that the power is distributed unequally in TMT (Finkelstein, 1992; Sperber and Linder, 2016). Therefore, we turn now to an indicator of internal context: CEO–TMT power gap, which is defined as the size of the power gap between the CEO and other executives. We argue that the CEO–TMT power gap is a double-edged sword, which generates both positive (e.g., due to efficiency advantage) and negative (e.g., due to quality disadvantage) dynamics that moderate the relationship between the CEO/TMT intrapersonal functional diversity and adaptive firm performance.

We argue that the CEO–TMT power gap is harmful for voice behavior. Specifically, voice refers to a process in which an employee tries to persuade the leader to accept the members' opinions or suggestions. This means that voice is an interaction process in which the leader is both the object and the evaluator. Given a high power gap possibly creating a sense of control and overconfidence in one's own abilities (Anderson et al., 2012), leaders in power are often reluctant to accept suggestions from other executives. Subsequently, the premature closure of the decision process will lead to low-quality decisions and poor performance. Moreover, scholars have argued that voice may lead to the worse performance evaluation for the actors (Whiting et al., 2012) because voice can be perceived as a challenge to the leader, especially for the prohibitive voice (Burris, 2012; Liang et al., 2012). As a result, a need for loss reduction may lead low-power executives to keep silent in the decision-making process, and then lower the adaptive firm performance. That is, the quality advantage of decision-making brought by CEO intrapersonal functional diversity will decrease with the increase of the CEO–TMT power gap.

*Hypothesis 1a: CEO–TMT power gap negatively moderates the effect of CEO intrapersonal functional diversity on adaptive firm performance*.

On the other hand, the CEO–TMT power gap has a positive effect on improving the efficiency advantage of decision-making. According to Finkelstein (1992), power is the capacity of individual actors to exert their will. Thus, a greater CEO–TMT power gap means that CEO has the capacity to control the meeting process. Considering that people have a biological instinct to draw on the advantages and avoid disadvantages, at the same time, the interests of CEOs are generally aligned with those of TMT and the organization to which they belong. Therefore, CEOs are motivated to resolve dysfunctional conflicts to strengthen the efficiency of the TMT decision (Hobfoll, 2011). Conversely, a smaller CEO–TMT power gap may lead to an interminable and useless discussion and consequently, a reduced efficiency advantage may follow. In this case, the efficiency advantage of decision-making brought by TMT intrapersonal functional diversity (e.g., due to shared task mental models) will be suppressed by the CEO–TMT power gap. Meanwhile, the decreased voice behaviors and information integrations derived from the CEO–TMT power gap may further strengthen the quality disadvantage of decision-making brought by TMT intrapersonal functional diversity (e.g., due to limited knowledge base). Overall, we argue that the impact of TMT intrapersonal functional diversity on adaptive firm performance will become less positive as the CEO–TMT power gap increases.

*Hypothesis 1b: CEO–TMT power gap negatively moderates the effect of TMT intrapersonal functional diversity on adaptive firm performance*.

### External Context: The Moderating Role of Severity of Threat

We turn now to an indicator of external context: severity of threat. According to the Threat Rigidity Theory proposed by Staw et al. (1981), there are significant differences in multi-level psychological states of focus entities under the different sources or intensities of threat. Specifically, when the external threat is considered solvable to a team, cohesiveness, leadership support, and the pressure for uniformity are formulated accordingly. These phenomena are noted as “restriction of information” and “restriction of control.” Otherwise, neither cohesiveness nor a consensus is likely to follow if a threat is attributed to the internal resources or considered difficult to solve. This may then provide TMT with new information or the loosening of control. Because the actual effectiveness of CEO's power varies with the source and intensity of threats, we, therefore, argue that the moderation of intrapersonal functional diversity's effect on adaptive firm performance by the CEO–TMT power gap is itself moderated by the severity of threat.

First, on the source of the threat, it should be noted that the focus entity of this study is TMT. Therefore, threats such as turnover or violent conflict in the TMT should be attributed to internal sources, while natural disasters, social events, market competition occurring outside the TMT should be attributed to external sources. Because COVID-19 was chosen as the target threat event for this study, the corresponding threat situation is naturally attributed to external sources. Regarding the perception of whether the team can overcome the threat successfully, the concept of team efficacy could provide necessary explanations. Specifically, team efficacy is the belief among team members that the team has a common ability to successfully accomplish a particular task, and direct experiences are the most important sources of team efficacy (Bandura, 2012). That is, a successful experience can usually enhance team efficacy, while repeated failure experiences may reduce team efficacy. To our knowledge, in general, a successful experience in dealing with severe threats is rare for any TMTs. Thus, team efficacy in responding to threat is more likely to decline as the severity of threat increases. Afterward, the decreased leadership support may follow. Based on the above analysis, we argue that the moderating effect of the CEO–TMT power gap on the relationship between the intrapersonal functional diversity and the adaptive firm performance will be weakened as the severity of threat increases.

*Hypothesis 2a: Severity of threat has a negative higher-order moderating effect on the interaction of CEO intrapersonal functional diversity and CEO–TMT power gap on adaptive firm performance*.
*Hypothesis 2b: Severity of threat has a negative higher-order moderating effect on the interaction of TMT intrapersonal functional diversity and CEO–TMT power gap on adaptive firm performance*.

**Figure 1:**
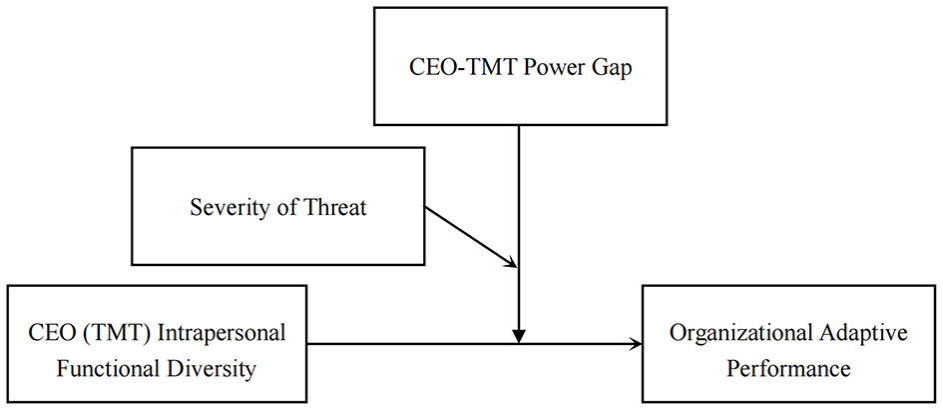
Theoretical method.

The theoretical model of this paper is shown in Figure 1.

## Materials and Methods

### Sample and Methods

To ensure the occurrence of the COVID-19 breakout, a landmark crisis attributed to external resources, which had brought more threat over chance for TMTs, we sampled the severely affected industries, for example, hotel and restaurant, health and social work, water conservancy and environment, transportation, entertainment, apparel, and automobile manufacturing industries. The magnitude of the COVID-19 outbreak on industries was measured by the first quarter-on-first quarter operating revenue growth. Having selected a set of industries, we identified all the firms in those industries from China's A-share listed companies for our research period 2020. By excluding ST and ^*^ST enterprises, a total number of 270 observations (firm-year) with complete data were left. All the data used in this paper are from the China Stock Market & Accounting Research (CSMAR) database.

### Measures

#### Adaptive Firm Performance

Adaptive firm performance refers to a positive change in firm performance. It is known that team adaption research is mainly conducted in experimental situations, and adaptive performance is generally calculated by the difference in performance between the two adjacent time points. For example, scholars (Mathieu et al., 2000; Ellis, 2006; Pearsall et al., 2010) evaluate the performance level at three time points: before the crisis breakout (T_1_), before adaptive training (T_2_), and after adaptive training (T_3_). Adaptive performance could be obtained by the subtraction of the performance in T_2_ from the performance in T_3_. Using the same logic, we calculated adaptive firm performance by the subtraction of the Return on Equity (ROE) in the first quarter of 2020 (Q1/2020) from the ROE in the fourth quarter of 2020 (Q4/2020). To help ensure the robustness of the results, return on assets (ROA) was used as a new approach to evaluate the adaptive firm performance, and the consistent results indicate that all conclusions are stable and reliable.

#### Intrapersonal Functional Diversity

Intrapersonal functional diversity refers to the breadth of member's functional experience (Cannella et al., 2008). To measure intrapersonal functional diversity at both individual and team levels, we first divided the executive's functional background into six categories (R&D and design; production; marketing; accounting and finance; management; and law). Then, a revised approach of Bunderson and Sutcliffe (2002) was adopted to measure the individual intrapersonal functional diversity of all the executives (including CEO). Individual intrapersonal functional diversity was calculated as $1-\sum {P_{j}}^{2}$, whereas *P*_*j*_ is the proportion of the total tenure of a functional background in one member's entire career. According to the recommendations by Cannella et al. (2008), we weighted each executive's functional areas equally because it is impossible for us to figure out the tenure of each functional area from annual reports. In simple terms, *P*_*j*_ is the reciprocal of the number of one member's functional areas.

#### CEO–TMT Power Gap

The CEO–TMT power gap was calculated by the difference between the CEO's relative power and the average relative power of the other executives. According to the recommendations by Finkelstein (1992) and the specific situations of China's A-share listed companies, five indexes were ultimately selected to calculate the executives' relative power. The five indexes are as follows: (1) the ratio of one's salary to the highest salary in the TMT, (2) the ratio of the number of one's official titles to the largest number of official titles in the TMT, (3) the ratio of one's shares to the highest shares in the TMT, (4) the ratio of one's educational level (1 = “senior high school or below,” 2 = “junior college,” 3 = “bachelor,” 4 = “master,” and 5 = “doctor”) to the highest educational level in the TMT, and (5) a CEO would be rated 2, 1.5, 1.25, 1 when he or she is also the chairman, vice chairman, director, or non-board member. Similarly, an executive (non-CEO) would be rated as 0.75, 0.5, 0.25, or 0 when he or she is also the chairman, vice chairman, director, or non-board member. Having calculated five indexes, a principal component factor analysis of all the five indexes was conducted to assess each executive's relative power. The factors with eigenvalues >1 were extracted using an oblique rotation method. Then, the relative power of an executive and the CEO–TMT power gap were calculated afterward. To help ensure the robustness of our results, the difference of relative power between a CEO and the highest executive (non-CEO) was selected as a new measurement of the CEO–TMT power gap, and the consistent results indicate that all conclusions are stable and reliable.

#### Severity of Threat

Severity of threat refers to the magnitude of the damage that an enterprise suffered from a crisis. Multiple anti-epidemic measures like enterprises stopped, work was put on hold, and schools closed have been applied since Wuhan was locked down on January 23, 2020. These measures subsequently bring a significant threat toward enterprise productions and operations. Because the severity of threat varies significantly across enterprises, we adopted an objective indicator: the quarter-on-quarter growth rate. Specifically, we calculated the severity of threat by the subtraction of the average ROE in Q1/2018 and Q1/2019 from the ROE in Q1/2020. The average ROE was used to reduce the bias caused by single-year outliers (Cannella et al., 2008). Noticing that the impacts of COVID-19 last longer, we calculated the severity of threat by the subtraction of the average ROE in Q1-Q2/2018 and Q1-Q2/2019 from the ROE in Q1-Q2/2020, and the consistent results indicated that the conclusions are stable and reliable.

#### Control Variables

The control variables used in this article are considered to be related to the organizational outcomes, and range from different aspects, including individual, team, organizational, and industrial level, especially, including CEO's age, gender, educational level, TMT size, average TMT tenure, TMT background diversity (i.e., functional diversity, educational diversity, tenure diversity, gender diversity, and age diversity), firm age (total years since IPO), and firm size (the natural log of the number of employees). Moreover, considering that the performance may vary across industries, the industry fixed effects were controlled in this study.

## Results

### Descriptive Statistics

Table 1 shows descriptive statistics and correlations. The intercorrelations among multiple TMT diversity variables are equal to or <0.21, this means that they can be included in a regression model (Carpenter and Fredrickson, 2001). Moreover, the correlation coefficients among core explanatory variables are equal to or <0.39, and the low variable inflation factor (VIF) scores (the maximum value 2.61 is well below the recommended cutoff of 10) suggest that multicollinearity was not a significant problem (Cannella et al., 2008).

**Table 1:** Descriptive statistics and pearson correlations.

**Variables**	**Mean**	**SD**	**1**	**2**	**3**	**4**	**5**
1. TMT intrapersonal functional diversity	0.55	0.06	–				
2. CEO intrapersonal functional diversity	0.57	0.09	0.39^***^	–			
3. CEO-TMT power gap	0.40	0.12	−0.07	0.22^***^	–		
4. Severity of threat	0.01	0.02	0.07	0.06	−0.06	–	
5. CEO gender	1.09	0.29	−0.09	−0.02	−0.01	0.01	–
6. CEO age	51.34	6.31	0.00	0.04	0.14^**^	−0.04	0.02
7. CEO educational level	3.38	0.91	−0.03	−0.04	0.19^***^	−0.05	−0.05
8. TMT size	5.73	1.60	0.07	0.02	0.08	0.01	−0.10
9. TMT average tenure	51.66	22.06	−0.11^*^	0.05	0.03	0.10	−0.02
10. TMT average age	47.91	3.61	0.09	0.03	−0.11^*^	0.12^**^	−0.03
11. TMT average educational level	3.29	0.51	−0.02	−0.03	−0.07	−0.05	−0.10
12. TMT tenure diversity	28.14	18.86	−0.14^**^	−0.03	−0.05	0.02	−0.01
13. TMT gender diversity	0.22	0.19	−0.16^**^	−0.10^*^	0.05	−0.01	0.38^***^
14. TMT age diversity	6.26	2.40	−0.02	0.06	0.09	−0.01	−0.10^*^
15. TMT educational diversity	0.65	0.29	−0.02	−0.04	0.08	0.107^*^	0.11^*^
16. TMT functional diversity	0.58	0.14	−0.08	0.06	0.12^**^	−0.06	0.06
17. Firm size	7.45	1.14	0.03	0.04	−0.13^**^	0.02	0.08
18. Firm age	9.04	7.14	−0.11^*^	0.00	−0.20^***^	0.09	0.02
19. Adaptive firm performance	0.05	0.10	0.06	0.04	0.07	−0.10	−0.08
**Variables**	**6**	**7**	**8**	**9**	**10**	**11**	**12**
6. CEO age	–						
7. CEO educational level	0.06	–					
8. TMT size	0.02	0.11^*^	–				
9. TMT average tenure	0.11^*^	0.05	−0.07	–			
10. TMT average age	0.58^***^	0.12^**^	0.06	0.24^***^	–		
11. TMT average educational level	0.10^*^	0.70^***^	0.02	0.01	0.07	–	
12. TMT tenure diversity	0.09	0.14^**^	0.11^*^	0.52^***^	0.13^**^	0.14^**^	–
13. TMT gender diversity	0.00	0.03	−0.02	−0.04	−0.14^**^	−0.04	−0.03
14. TMT age diversity	0.22^***^	−0.09	−0.04	−0.03	−0.05	−0.05	0.00
15. TMT educational diversity	0.06	−0.18^***^	0.18^***^	−0.06	0.08	−0.21^***^	−0.04
16. TMT functional diversity	−0.01	−0.04	0.13^**^	0.02	−0.16^***^	−0.02	−0.05
17. Firm size	0.13^**^	0.05	0.21^***^	−0.02	0.24^***^	0.15^**^	0.09
18. Firm age	0.11^*^	0.18^***^	0.04	0.28^***^	0.27^***^	0.24^***^	0.48^***^
19. Adaptive firm performance	0.06	−0.04	0.08	0.02	0.10	−0.05	−0.14^**^
**Variables**	**13**	**14**	**15**	**16**	**17**	**18**	**19**
13. TMT gender diversity	–						
14. TMT age diversity	0.09	–					
15. TMT educational diversity	0.00	0.21^***^	–				
16. TMT functional diversity	0.05	0.13^**^	0.03	–			
17. Firm size	−0.09	−0.12^*^	0.00	−0.04	–		
18. Firm age	−0.03	−0.12^**^	−0.05	−0.17^***^	0.30^***^	–	
19. Adaptive firm performance	−0.14^**^	−0.08	0.03	0.02	0.20^***^	−0.05	–

*n = 270,*

* *p < 0.1,*

** *p < 0.05,*

*** *p < 0.01*.

All regression results are presented in Table 2. Specifically, Model 1 is the baseline model, including only the control variables, then the explanatory variables are included in Model 2 to test the main effect. Models 3 and 4 presented the second-order moderating effect of the CEO–TMT power gap, while Models 5 and 6 presented the higher-order moderating effect of the severity of threat.

**Table 2:** The results of hierarchical regression analysis for adaptive firm performance.

**Variables**	**M1**	**M2**	**M3**	**M4**	**M5**	**M6**
CEO intrapersonal functional diversity		0.01	0.00	0.00	0.00	0.00
TMT intrapersonal functional diversity		−0.01	0.00	0.00	0.00	0.00
CEO-TMT power gap			0.01	0.01	0.01	0.01^*^
Severity of threat			−0.02^**^	−0.02^**^	−0.02^**^	−0.02^**^
CEO intrapersonal functional diversity × CEO-TMT power gap			−0.01		−0.01	
TMT intrapersonal functional diversity × CEO-TMT power gap				−0.01		−0.01
CEO intrapersonal functional diversity × Severity of threat					0.00	
TMT intrapersonal functional diversity × Severity of threat						0.01
CEO-TMT power gap × Severity of threat					−0.02^**^	−0.02
CEO intrapersonal functional diversity × CEO-TMT power gap × Severity of threat					−0.03^**^	
TMT intrapersonal functional diversity × CEO-TMT power gap × Severity of threat						−0.01
CEO gender	−0.09	−0.09	−0.03	−0.04	−0.04	−0.04^*^
CEO age	0.02	0.02	0.00	0.00	0.00	0.00
CEO educational level	0.02	0.02	−0.01	−0.01	−0.01	−0.01
TMT size	0.06	0.06	0.00	0.00	0.00	0.00
TMT average tenure	0.10	0.09	0.00	0.00	0.00	0.00
TMT average age	−0.01	−0.01	0.00	0.00	0.00	0.00
TMT average educational level	−0.05	−0.05	0.00	0.00	0.00	0.00
TMT tenure diversity	−0.14^*^	−0.13^*^	−0.00^*^	−0.00^*^	−0.00^**^	−0.00^**^
TMT gender diversity	−0.06	−0.06	−0.03	−0.03	−0.02	−0.02
TMT age diversity	−0.02	−0.02	0.00	0.00	0.00	0.00
TMT educational diversity	−0.02	−0.02	−0.01	−0.01	−0.01	−0.01
TMT functional diversity	0.01	0.01	0.00	0.00	0.01	0.00
Firm size	0.24^***^	0.24^***^	0.02^***^	0.02^***^	0.02^***^	0.02^***^
Firm age	Included	Included	Included	Included	Included	Included
Industry fixed effects	−0.09	−0.09	0.00	0.00	0.00	0.00
Constant	−0.07	−0.07	−0.12	−0.12	−0.15	−0.13
Adjusted R^2^	0.25	0.25	0.27	0.27	0.30	0.29
F	3.50^***^	3.20^***^	3.21^***^	3.25^***^	3.26^***^	3.06^***^

*n = 270,*

* *p < 0.1,*

** *p < 0.05,*

*** *p < 0.01*.

### Hypotheses Testing

The results presented in Model 2 are consistent with our viewpoint that the main effects of CEO or TMT intrapersonal functional diversity on adaptive firm performance are not significant (though no hypotheses were set).

Hypothesis H1a predicts a negative interaction between CEO intrapersonal functional diversity and CEO–TMT power gap affecting adaptive firm performance. Model 3 shows that the two-way interaction coefficient is negative but non-significant (β = −0.01, *p* > 0.05), and thus H1a is not supported.

Hypothesis H1b predicts a negative interaction between TMT intrapersonal functional diversity and the CEO–TMT power gap affecting adaptive firm performance. Model 4 shows that the two-way interaction coefficient is negative but non-significant (β = −0.01, *p* > 0.05), and thus H1b is not supported.

Hypothesis H2a predicts a negative interaction among CEO intrapersonal functional diversity, the CEO–TMT power gap, and the severity of threat affecting adaptive firm performance. Model 5 shows that the three-way interaction coefficient is negative and significant (β = −0.03, *p* < 0.05), and thus H2a is supported.

Hypothesis H2b predicts a negative interaction among TMT intrapersonal functional diversity, the CEO–TMT power gap, and the severity of threat affecting adaptive firm performance. Model 6 shows that the three-way interaction coefficient is negative but non-significant (β = −0.01, *p* > 0.05), and thus H2b is not supported.

**Figure 2:**
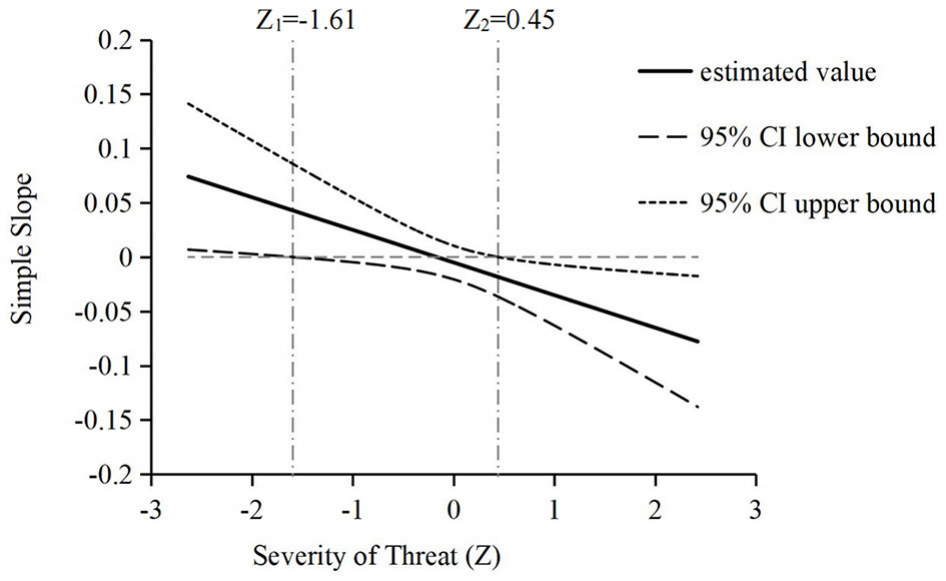
Moderating effect of severity of threat on the interaction between CEO-TMT power gap and CEO intrapersonal functional diversity.

Considering that the severity of threat is a quantitative dimension, the Johnson–Neyman (J–N) technique is a better method for simple effects analyses than the pick-a-point method. This is because the J–N technique could be used to ascertain where on the severity of threat continuum the two-way interaction between the CEO intrapersonal functional diversity and CEO–TMT power gap is significantly not equal to 0 (Hayes and Matthes, 2009). As shown in Figure 2, a two-way interaction between the CEO intrapersonal functional diversity and CEO–TMT power gap is significantly positive, significantly negative, and nonsignificant when the severity of threat is <−1.61,>0.45, and located between −1.61 and 0.45, respectively.

To ascertain where on a two-way interaction between the severity of threat and CEO–TMT power gap continuum, the simple effect of CEO intrapersonal functional diversity is significantly not equal to 0. We first divide the severity of threat into low- (<−1.61) and high-score group (>0.45), and then the J-N method was used in both groups respectively to ascertain where on the CEO-TMT power gap continuum (i.e., under conditions in which the severity of threat has been fixed) the simple effect of CEO intrapersonal functional diversity is significantly not equal to 0. The results show that: (1) when the severity of threat is <−1.61 and the CEO–TMT power gap is >1.49, the simple effect of CEO intrapersonal functional diversity on adaptive firm performance is positive and significant, (2) when the severity of threat is >0.45 and the CEO–TMT power gap is >1.67, the simple effect of CEO intrapersonal functional diversity on adaptive firm performance is negative and significant, (3) when the CEO–TMT power gap is less than the mean, regardless of the value the severity of threat takes, the simple effect of CEO intrapersonal functional diversity on adaptive firm performance is non-significant.

## Discussion

This study is designed to determine the effects of intrapersonal functional diversity (at both CEO and TMT levels) on adaptive firm performance. Scholars have long argued that diversification is associated with its adaptation to a dynamic environment (Cannella et al., 2008; Qian et al., 2012; Martins, 2020) while empirical studies have reached inconsistent results regarding the TMT functional diversity-firm performance relationship as Hambrick and Mason (1984) proposed the Upper Echelons Theory. A valuable explanation is that the abovementioned relationship depends on the conceptualization of functional diversity (e.g., dominating functional diversity and intrapersonal functional diversity) and also on the match between the internal structure and the external demands (Cannella et al., 2008). To this end, we explored the impact of CEO/TMT intrapersonal functional diversity on adaptive firm performance, and the moderating roles of the CEO–TMT power gap and severity of threat. This study contributes TMT diversity, power distribution, and team adaptation research in several ways.

This study extends the work of Cannella et al. (2008) not only by exploring the relationship between intrapersonal functional diversity and firm performance at both CEO and TMT levels but also by exploring the relationship in a threat situation that is different from general situations to a large extent. In contrast with the previous studies, TMT intrapersonal functional diversity positively affects firm performance (Bunderson and Sutcliffe, 2002; Cannella et al., 2008), no significant main effect of intrapersonal functional diversity on adaptive firm performance was found at both CEO and TMT levels. A possible explanation is that the CEO/TMT intrapersonal functional diversity carries both costs and benefits and may exhibit different or even opposite effects on the adaptive firm performance under different internal and external contexts. This explanation was confirmed in the subsequent results that there was a negative and significant three-way moderating effect among intrapersonal functional diversity, the CEO–TMT power gap, and the severity of threat at the CEO level but not at the TMT level.

Our study also contributes to the field of team power distribution. Previous studies indicated that the unequal power distribution is likely to trigger dysfunctional conflict and impede information sharing, which leads to a negative impact on organizational outcomes afterward (Eisenhardt and Bourgeois, 1988; Patel and Cooper, 2014). However, the impact of TMT power distribution on organizational outcomes may depend on the sociocultural values in which it operates (Vuong et al., 2020). For example, in countries with high power distance and high collectivism, a certain power gap between superiors and subordinates is considered to be in line with social norms (Zhang and Zhang, 2016), and recent studies in Chinese culture have found that TMT power centralization has a positive impact on firm performance (Cao et al., 2015; Zhang and Zhang, 2016). Overall, scholars have converged on the view that power distribution is a double-edged sword, but surprisingly there is little empirical research exploring the contextual boundaries of the effect of power distribution on organizational outcomes, especially for CEO power centralization (Bunderson, 2003). Our evidence is consistent with that of Zhang et al. (2020) who argues that a powerful CEO is an important factor in predicting a strategic change but will lead to differentiated performance according to the prior performance. A possible explanation is that limited resources and the multi-level threat rigidity effects may inhibit the recovery or improvement of the poor firm performance during the early stages of the crisis. Therefore, if the nature of the external environment (e.g., severity of threat) is ignored, it is not surprising to get mixed results in the study of TMT power distribution and also the study of TMT functional diversity.

Another contribution of this study is that we expand team adaptation research using the multilevel perspective. Existing studies on antecedents of team adaptation mainly considered the factors at the team level, whereas the factors at other levels and the possible relationships among them were ignored (Maynard et al., 2015). This study is among the first to examine the logical assumption of the team adaptation theory that the characteristics of a leader are more valuable than team average characteristics in predicting adaptive firm performance. Surprisingly, the idea was confirmed by a moderating effect but not by the main effect. That is, the moderating effects of the CEO–TMT power gap and severity of threat on intrapersonal functional diversity in predicting adaptive firm performance between the CEO and TMT levels is significantly different, but there is no difference in the main effects. Considering that leader support may vary across different situations, a significant part of the variations in this study can be explained by contextual differences.

We also expand team adaptation research by considering the nature (e.g., severity of threat) of triggers in a field study. Specifically, existing studies on team adaptation are mainly conducted in laboratory settings and mostly focused on military tasks. Since a significant difference between the laboratory situation and the actual business activities in daily life, researchers are calling for a field survey to improve the external validity of team adaptation research (Rosen et al., 2011). Moreover, note that teams are complex, adaptive, and dynamic systems, they exist in and interact with the context (Ilgen et al., 2005). Therefore, a growing body of literature holds the perspective that the effectiveness of team adaptation antecedents depends on the nature of adaptation trigger faced by the team (Baard et al., 2013; Maynard et al., 2015; Christian et al., 2017). According to the Threat Rigidity Theory, we focused on two dimensions of the nature of threat: source and severity. As expected by the assumptions in this study, the results confirm that the interaction of CEO intrapersonal functional diversity and the CEO–TMT power gap varies significantly across threat situations. Therefore, we argue that an efficient response to a trigger brought by TMT characteristic composition and power centralization is not necessarily an effective strategy while the situation is unknown. This is because the adjustments of the team processes in pursuit of continuous improvement may not always be functional, and the costs of the adjustments may lower the efficiency and the quality of TMT decision-making. In fact, highly performing teams can maintain a high functionality by simply repeating past behavioral patterns unless the situation changes substantially (Gevers et al., 2015).

A concern about the stability of the conclusions is whether different results can be obtained in situations without external environmental threats such as COVID-19? To increase the robustness of this research conclusion, a supplementary analysis different from the analyses in the measures section was conducted using a new data set of the 270 enterprises in a non-threatening situation. Specifically, the data set includes CEO/TMT intrapersonal functional diversity for 2017, the mean of ROA for 2018 and 2019, and a series of control variables for 2017. The results showed that the main effect of CEO intrapersonal functional diversity on firm performance is positive but not significant (β = 0.101, *p* > 0.10). However, TMT intrapersonal functional diversity has a significant positive impact on firm performance (β = 0.157, *p* < 0.05) and explains an additional 2.2% of the variation in firm performance. As expected, these results are consistent with those of Bunderson and Sutcliffe (2002) and Cannella et al. (2008), providing further support for our view. That is, firms have different demands for CEO intrapersonal functional diversity or TMT intrapersonal functional diversity in different contexts.

## Practical Implications

This study offers insights for managerial practice. Practitioners have been struggling to identify and utilize the capacities possessed by the TMT necessary for addressing contextual challenges. TMT demographic composition, especially for TMT functional diversity, has been considered as an important antecedent of various organizational outcomes including adaptive performance. This study indicated that the level of the CEO intrapersonal functional diversity is considered to be more valuable than that of TMT average intrapersonal functional diversity in predicting adaptive firm performance. Furthermore, this study points out an important caveat to the benefit of CEO intrapersonal functional diversity: the recovery or improvement of the firm performance in severe threat conditions requires different levels of both knowledge depth and CEO power centralization than those needed in low threat conditions. Therefore, organizations with challenging environments (i.e., new crisis episodes may break out at any moment) should assign a powerful CEO with narrower functional experiences. This is possible because a deeper knowledge structure of low intrapersonal functional diversity can provide more chances for recombining the existing knowledge base. Meanwhile, power centralization allows the CEO to control the formulation and execution processes of the decision. This allows the CEO to maintain a balance between decision quality and decision efficiency, and both of them are important to crisis management. Consequently, TMT effectiveness and excellent firm performance will follow. On the other hand, a powerful CEO with high intrapersonal functional diversity should be assigned in industries that are characterized as stable. This is possible because the inclusive atmosphere, created by the CEO with broadened functional experiences, may encourage other executives to engage in more voice behaviors or constructive debates, and thus contributing to firm performance.

## Limitations

Like most empirical studies, this study has several limitations. Since the scarcity and sensitivity of top managers, it is extremely difficult to conduct a questionnaire survey on the TMT. Therefore, based on the research paradigm of the Upper Echelons Theory, this paper gathered a second-hand data set for hypothesis testing similar to previous studies, which leads to a common limitation in the TMT research field. That is, there is a serious logical leap in the hypothesis formulation process, which prevents us from measuring a large number of team processes and emergent states (e.g., trust, transactive memory system, shared mental model, and voice behavior), and thus leaving the mechanism of CEO intrapersonal functional diversity on adaptive firm performance still in the black box. For example, we assume that CEO–TMT power gap has a negative moderating effect on the relationship between CEO/TMT intrapersonal functional diversity and adaptive firm performance because a larger CEO–TMT power gap makes voice behavior less likely. However, we did not empirically test the relationship between the CEO–TMT power gap and voice behavior but made hypotheses based on an implicit inference. Therefore, future studies can unpack the black box by adopting a multiwave cross-sectional questionnaire survey.

Second, the present study focused on organizations, which are severely affected due to the COVID-19 pandemic. However, various threat situations such as environmental breakdown and climate crisis may be different from the threat caused by the COVID-19 in various aspects (Vuong, 2021), but this article provides no information on them. In addition, although the findings provide implications for crisis management, the effect of the intrapersonal functional diversity (at both individual and team levels) on firm performance in general situations remains uncertain. Future studies can explore whether and how the impacts of intrapersonal functional diversity vary across the different types, nature, source, and severity of threat situations.

Third, the present study adopted five subindicators to measure individual relative power but ignored the other subindicators suggested by Finkelstein (1992) because the corresponding information was usually not shown in the enterprise report. Therefore, more subindicators are required to measure the individual relative power, and the conclusions of this study need to be tested in future studies.

## Conclusions

Top management team scholars (Bunderson and Sutcliffe, 2002; Cannella et al., 2008; Qian et al., 2012; Hambrick et al., 2015) have generally believed that a mixed relationship between TMT functional diversity and firm performance arises from different conceptualizations of diversity, as well as ignoring the effects of both internal and external contexts. Based on the abovementioned consideration, this study examined the relationship among intrapersonal functional diversity, the CEO–TMT power gap, the severity of threat, and adaptive firm performance. The findings suggest that CEO intrapersonal functional diversity is more valuable than TMT intrapersonal functional diversity in predicting adaptive firm performance. Moreover, the relationship between CEO intrapersonal functional diversity and adaptive firm performance depends on both the CEO–TMT power gap and the severity of threat. Overall, the TMT led by a powerful CEO with narrower functional experiences is more adaptive to a severe external threat, while the TMT led by a powerful CEO with broader functional experiences is more adaptive to a mild external threat. However, no matter high or low on intrapersonal functional diversity, the powerless CEO may not be capable of leading the organization out of the crisis.

## Data Availability Statement

The original contributions presented in the study are included in the article/supplementary material, further inquiries can be directed to the corresponding author/s.

## Author Contributions

All authors listed have made a substantial, direct, and intellectual contribution to the work and approved it for publication.

## Funding

This research was supported by the Ministry of education of Humanities and Social Science project of China (17YJA630020).

## Conflict of Interest

The authors declare that the research was conducted in the absence of any commercial or financial relationships that could be construed as a potential conflict of interest.

## Publisher's Note

All claims expressed in this article are solely those of the authors and do not necessarily represent those of their affiliated organizations, or those of the publisher, the editors and the reviewers. Any product that may be evaluated in this article, or claim that may be made by its manufacturer, is not guaranteed or endorsed by the publisher.

## References

[B1] Anderson C. John O. P. Keltner D. (2012). The personal sense of power. J. Pers. 80, 313–344. 10.1111/j.1467-6494.2011.00734.x 21446947

[B2] Baard S. K. Rench T. A. Kozlowski S. W. J. (2013). Performance adaptation. J. Manage. 40, 48–99. 10.1177/0149206313488210

[B3] Bandura A (2012). On the functional properties of perceived self-efficacy revisited. J. Manage. 38, 9–44. 10.1177/014920631141060612675397

[B4] Bunderson J (2003). Team member functional background and involvement in management teams: direct effects and the moderating role of power centralization. Acad. Manage. J. 46, 458–474. 10.5465/30040638

[B5] Bunderson J. S. Sutcliffe K. M. (2002). Comparing alternative conceptualizations of functional diversity in management teams: process and performance effects. Acad. Manage. J. 45, 875–893. 10.5465/3069319

[B6] Burke C. S. Stagl K. C. Salas E. Pierce L. Kendall D. (2006). Understanding team adaptation: a conceptual analysis and model. J. Appl. Psychol. 91, 1189–1207. 10.1037/0021-9010.91.6.1189 17100478

[B7] Burris E. R (2012). The risks and rewards of speaking up: managerial responses to employee voice. Acad. Manage. J. 55, 851–875. 10.5465/amj.2010.0562

[B8] Cannella A. A. Park J. H. Lee H. U. (2008). Top management team functional background diversity and firm performance: examining the roles of team member colocation and environmental uncertainty. Acad. Manage. J. 51, 768–784. 10.5465/AMJ.2008.33665310

[B9] Cao J. Yang B. Yang B. Y. (2015). Top management team power distribution and firm performance: evidence from listed firms in shanghai and shenzhen stock exchange. Sci. Sci. Manage. S. T. 36, 135–145.

[B10] Carmeli A. Paulus P. B. (2015). CEO ideational facilitation leadership and team creativity: the mediating role of knowledge sharing. J. Creat. Behav. 49, 53–75. 10.1002/jocb.5925855820

[B11] Carpenter M. A. Fredrickson J. W. (2001). Top management teams, global strategic posture, and the moderating role of uncertainty. Acad. Manage. J. 44, 533–546. 10.5465/3069368

[B12] Christian J. S. Christian M. S. Pearsall M. J. Long E. C. (2017). Team adaptation in context: An integrated conceptual model and meta-analytic review. Organ. Behav. Hum. Decis. Process. 140, 62–89. 10.1016/j.obhdp.2017.01.003

[B13] Collins C. J. Smith K. G. (2006). Knowledge exchange and combination: the role of human resource practices in the performance of high-technology firms. Acad. Manage. J. 49, 544–560. 10.5465/amj.2006.21794671

[B14] Cooper D. Patel P. C. Thatcher S. M. B. (2014). It depends: environmental context and the effects of faultlines on top management team performance. Organizat. Sci. 25, 633–652. 10.1287/orsc.2013.0855

[B15] Dane E (2010). Reconsidering the trade-off between expertise and flexibility: a cognitive entrenchment perspective. Acad. Manage. Rev. 35, 579–603. 10.5465/AMR.2010.53502832

[B16] Eisenhardt K. Bourgeois L. J. (1988). Politics of strategic decision making in high-velocity environments: toward a midrange theory. Acad. Manage. J. 31, 737–770. 10.5465/256337

[B17] Ellis A. P. J (2006). System breakdown: the role of mental models and transactive memory in the relationship between acute stress and team performance. Acad. Manage. J. 49, 576–589. 10.5465/amj.2006.21794674

[B18] Finkelstein S (1992). Power in top management teams: dimensions, measurement, and validation. Acad. Manage. J. 35, 505–538. 10.5465/256485 10120413

[B19] Georganta E. Kugler K. G. Reif J. A. M. Brodbeck F. C. (2021). Diving deep into team adaptation: how does it really unfold over time? Group Dynam. Theory Res. Pract. 25, 137–151. 10.1037/gdn0000133

[B20] Gevers J. M. P. Uitdewilligen S. Passos A. M. (2015). Dynamics of team cognition and team adaptation: introduction to the special issue. Euro. J. Work Organization. Psychol. 24, 645–651. 10.1080/1359432X.2015.1065251

[B21] Gino F. Argote L. Miron-Spektor E. Todorova G. (2010). First, get your feet wet: the effects of learning from direct and indirect experience on team creativity. Organ. Behav. Hum. Decis. Process. 111, 102–115. 10.1016/j.obhdp.2009.11.002

[B22] Hambrick D. C (2007). Upper echelons theory: an update. Acad. Manage. Rev. 2 334–343. 10.5465/amr.2007.24345254

[B23] Hambrick D. C. Humphrey S. E. Gupta A. (2015). Structural interdependence within top management teams: a key moderator of upper echelons predictions. Strateg. Manage. J. 36, 449–461. 10.1002/smj.223025855820

[B24] Hambrick D. C. Mason P. A. (1984). The organization as a reflection of its top managers. Acad. Manage. Rev. 9, 193–206. 10.2307/258434

[B25] Hayes A. F. Matthes J. (2009). Computational procedures for probing interactions in OLS and logistic regression: SPSS and SAS implementations. Behav. Res. Methods. 41, 924–936. 10.3758/BRM.41.3.924 19587209

[B26] Hobfoll S. E (2011). Conservation of resource caravans and engaged settings. J. Occup. Organ. Psychol. 84, 116–122. 10.1111/j.2044-8325.2010.02016.x

[B27] Ilgen D. R. Hollenbeck J. R. Johnson M. Jundt D. (2005). Teams in organizations: from input-process-output models to IMOI models. Annu. Rev. Psychol. 56, 517–543. 10.1146/annurev.psych.56.091103.070250 15709945

[B28] Klein K. J. Kozlowski S. W. J. (2016). From Micro to Meso: critical steps in conceptualizing and conducting multilevel research. Organ. Res. Methods. 3, 211–236. 10.1177/109442810033001

[B29] Li Y. Li N. Harris T. B. Li J. Y. Guo J. Z. (2018). A network view of advice-giving and individual creativity in teams: a brokerage-driven, socially perpetuated phenomenon. Acad. Manage. J. 61, 2210–2229. 10.5465/amj.2016.0212

[B30] Liang J. Farh C. I. C. Farh J.-L. (2012). Psychological antecedents of promotive and prohibitive voice: a two-wave examination. Acad. Manage. J. 55, 71–92. 10.5465/amj.2010.0176

[B31] Mannucci P. V. Yong K. (2018). The differential impact of knowledge depth and knowledge breadth on creativity over individual careers. Acad. Manage. J. 61, 1741–1763. 10.5465/amj.2016.0529

[B32] Marks M. A. Zaccaro S. J. Mathieu J. E. (2000). Performance implications of leader briefings and team-interaction training for team adaptation to novel environments. J. Appl. Psychol. 85, 971–986. 10.1037/0021-9010.85.6.971 11125660

[B33] Martins L. L (2020). Strategic diversity leadership: the role of senior leaders in delivering the diversity dividend. J. Manage. 46, 1191–1204. 10.1177/0149206320939641

[B34] Mathieu J. E. Heffner T. S. Goodwin G. F. Salas E. Cannon-Bowers J. A. (2000). The influence of shared mental models on team process and performance. J. Appl. Psychol. 85, 273–283. 10.1037/0021-9010.85.2.273 10783543

[B35] Maynard M. T. Kennedy D. M. Sommer S. A. (2015). Team adaptation: a fifteen-year synthesis (1998–2013) and framework for how this literature needs to adapt going forward. Euro. J. Work Organization. Psychol. 24, 652–677. 10.1080/1359432X.2014.1001376

[B36] Patel P. Cooper D. (2014). Structural power equality between family and non-family tmt members and the performance of family firms. Acad. Manage. J. 57, 1624–1649. 10.5465/amj.2012.0681

[B37] Pearsall M. J. Ellis A. P. Bell B. S. (2010). Building the infrastructure: the effects of role identification behaviors on team cognition development and performance. J. Appl. Psychol. 95, 192–200. 10.1037/a0017781 20085416

[B38] Qian C. Cao Q. Takeuchi R. (2012). Top management team functional diversity and organizational innovation in China: the moderating effects of environment. Strateg. Manage. J. 34, 110–120. 10.1002/smj.199325855820

[B39] Rosen M. A. Bedwell W. L. Wildman J. L. Fritzsche B. A. Salas E. Burke C. S. (2011). Managing adaptive performance in teams: guiding principles and behavioral markers for measurement. Hum. Resour. Manage. Rev. 21, 107–122. 10.1016/j.hrmr.2010.09.003

[B40] Sperber S. Linder C. (2016). The impact of top management teams on firm innovativeness: a configurational analysis of demographic characteristics, leadership style and team power distribution. Rev. Manager. Sci. 12, 285–316. 10.1007/s11846-016-0222-z

[B41] Staw B. Sandelands L. Dutton J. (1981). Threat rigidity effects in organizational behavior: a multilevel analysis. Admin. Sci. Q. 26, 501–524. 10.2307/2392337

[B42] Tajfel H. Turner J. C. (1986). The social identity theory of intergroup behavior. Psychol. Intergroup Relat. 13, 7–24.

[B43] Vuong Q.-H. Bui Q.-K. La V.-P. Vuong T.-T. Nguyen V.-H. T. Ho M.-T.. (2018). Cultural additivity: behavioural insights from the interaction of Confucianism, Buddhism and Taoism in folktales. Palgrave Commun. 4:2. 10.1057/s41599-018-0189-2

[B44] Vuong Q. H (2021). The semiconducting principle of monetary and environmental values exchange. Econ. Bus. Lett. 10, 284–290. 10.17811/ebl.10.3.2021.284-290

[B45] Vuong Q. H. Manh-Tung H. Nguyen H. K. T. Vuong T. T. Tran T. Hoang K. L.. (2020). On how religions could accidentally incite lies and violence: folktales as a cultural transmitter. Palgrave Commun. 6:3. 10.1057/s41599-020-0442-3

[B46] Vuong Q. H. Napier N. K. (2015). Acculturation and global mindsponge: an emerging market perspective. Int. J. Intercult. Relat. 49, 354–367. 10.1016/j.ijintrel.2015.06.003

[B47] Whiting S. W. Maynes T. D. Podsakoff N. P. Podsakoff P. M. (2012). Effects of message, source, and context on evaluations of employee voice behavior. J. Appl. Psychol. 97, 159–182. 10.1037/a0024871 21842973

[B48] Zhang J. J. Zhang Y. L. (2016). Chairman-CEO heterogeneity, power differential, rapport and organizational performance. Management World, 110-120+188. 10.19744/j.cnki.11-1235/f.2016.01.011

[B49] Zhang M. Lan H. L. Chen W. H. Zeng P. (2020). Research on the antecedent configuration and performance of strategic change. Manage. World. 36, 168–186. 10.19744/j.cnki.11-1235/f.2020.0145

